# Detection and Quantification of the Fragile X Mental Retardation Protein 1 (FMRP)

**DOI:** 10.3390/genes7120121

**Published:** 2016-12-09

**Authors:** Giuseppe LaFauci, Tatyana Adayev, Richard Kascsak, W. Ted Brown

**Affiliations:** NYS Institute for Basic Research, Staten Island, NY 10314, USA; tatyana.adayev@opwdd.ny.gov (T.A.); richard.kascsak@opwdd.ny.gov (R.K.); ted.brown@opwdd.ny.gov (W.T.B.)

**Keywords:** FXS, FMRP, western blot, ELISA, TR-FRET, DBS, newborn screening, capture immunoassays, FMRP expression

## Abstract

The final product of *FMR1* gene transcription, Fragile X Mental Retardation Protein 1 (FMRP), is an RNA binding protein that acts as a repressor of translation. FMRP is expressed in several tissues and plays important roles in neurogenesis, synaptic plasticity, and ovarian functions and has been implicated in a number of neuropsychological disorders. The loss of FMRP causes Fragile X Syndrome (FXS). In most cases, FXS is due to large expansions of a CGG repeat in *FMR1*—normally containing 6–54 repeats—to over 200 CGGs and identified as full mutation (FM). Hypermethylation of the repeat induces *FMR1* silencing and lack of FMRP expression in FM male. Mosaic FM males express low levels of FMRP and present a less severe phenotype that inversely correlates with FMRP levels. Carriers of pre-mutations (55–200 CGG) show increased mRNA, and normal to reduced FMRP levels. Alternative splicing of *FMR1* mRNA results in 24 FMRP predicted isoforms whose expression are tissues and developmentally regulated. Here, we summarize the approaches used by several laboratories including our own to (a) detect and estimate the amount of FMRP in different tissues, developmental stages and various pathologies; and (b) to accurately quantifying FMRP for a direct diagnosis of FXS in adults and newborns.

## 1. Introduction

Fragile X syndrome (FXS) is the most common inherited cause of intellectual disability and is frequently associated with autism. In the vast majority of cases, the syndrome is caused by the lack of, or extremely reduced expression of the Fragile X Mental retardation protein 1 (FMRP), the gene product of *FMR1*. FMRP is a RNA-binding protein that interacts with a large number of mRNAs and several proteins including Fragile X Related Proteins 1 and 2 (FXR1, FXR2) both sharing extensive homology to FMRP [1,2]. FXS is commonly caused by large expansions of a trinucleotides repeats (CGG) sequence (normally containing 6–44 repeats), increasing to over 200 CGG (full mutation (FM)), which leads to hypermethylation of the *FMR1* gene promoter and silences transcription. The FM allele is maternally inherited and has an approximate prevalence of 1 in 4000 in the North American population. The CGG repeat region is located in the 5′ untranslated (UTR) region of the *FMR1* open reading frame and in control individuals does not change in size upon transmission to the offspring. *FMR1* alleles with 45–54 repeats and with 55–200 repeats are classified as intermediate and pre-mutation (PM) alleles, respectively, and are unstable upon transmission. 

PM carriers have an estimated prevalence ranging from 1/151 to 1/209 for females and 1/430 to 1/468 for males [3,4]. Some individuals are classified as FM mosaic, because their cells carry both the FM and a PM allele (size mosaicism), or because a fraction of their cells carry unmethylated FM alleles (methylation mosaicism). PM alleles are highly unstable and may expand to the FM in one generation when transmitted by a female. PM carriers have normal or somewhat reduced FMRP levels and increased *FMR1* mRNA that is inefficiently translated. Although they usually have normal cognitive functions, some adult carriers have a variety of immune and psychiatric disorders, such as fibromyalgia, elevated symptoms of anxiety and depression, attention hyperactivity disorder, and a progressive age-related decline in executive function [5]. It has also been reported that in aged male carriers of PMs larger than 100 CGG there is a correlation between age and poor task performance on executive functions linked to inhibition and executive working memory [6]. In some ‘non-affected’ carriers, the presence of symptoms evocative of those observed in FXS has led to the hypothesis that they could be triggered by lower levels of FMRP [7,8]. Fragile X-associated primary ovarian insufficiency (FXPOI) and premature menopause develops in 20% to 25% of PM women. 

It is also estimated that approximately 30% of older male PM carriers and some female carriers develop a late onset condition known as Fragile X-associated tremor/ataxia syndrome (FXTAS) [9]. These individuals develop cerebella ataxia, kinetic tremor, psychiatric problems, cognitive decline, and Parkinsonism [10,11]. FXTAS and FXPOI are thought to be gain-of-function pathologies resulting either from over-expression of the PM mRNA or from cryptic polyglycine-containing toxic proteins produced by repeat associated non-AUG initiated (RAN) translation of the CGG repeats [12]. 

Since the lack of FMRP expression is the cause of FXS [13,14,15,16] and its reduced levels may play a role in determining some phenotypes associated with PM alleles [7], efforts to detect and quantify the protein have been undertaken by several laboratories using various approaches. In this review, we summarize the several methods, including those developed by our group that have been used to detect FMRP and quantify its expression in distinct FMR1 genotypes, tissues, developmental stages, and pathologies.

## 2. Western Blot

Western blotting, the electrophoretic transfer of proteins separated in polyacrylamide gels either onto a nitrocellulose or a polyvinylidene difluoride (PVDF) membrane [17], has been widely used with specific antibodies to establish the presence of specific proteins in cell extracts, and to estimate the protein’s size and relative abundance. The Western blot profile of FMRP was characterized by Mandel and colleagues [13] using mouse monoclonal antibody MAB2160 (Table 1), rose against a bacteria-expressed recombinant FMRP (clone C17). This antibody, also referred to as mAb1C3, and mAb1A has been employed in a large number of publications (MAB2160—Chemicon-International, Milwaukee, WI, USA). In a variety of control human cells, western blotting with this antibody has revealed four to five bands, ranging from 70- to 80-kDA. No immunostaining was detected using lymphoblastoid cell lines derived from FM males.

Oostra and colleagues raised anti-FMRP rabbit polyclonal antibodies [14] and used one of them, α765 (Table 1) to immunoprecipitate FMRP from lymphoblastoid cell lines derived from patients and controls. Western blot analysis of the immunoprecipitated material revealed four bands (67–74 kDA) in control samples and none in those from FXS male patients. Brown et al. used mAb7G1-1 (Table 1) [18] to immunoprecipitate Fmrp from mouse brain samples and showed in Western blots with MAB2160 the presence of at least three major bands [37]. The reactivity of the above antibodies with multiple proteins was explained by the presence of several mRNA isoforms—produced by alternative splicing of *FMR1*—found in human lymphoblastoid cells [14] and mouse tissues [19,20]. However, it has been reported that in *FMR1* KO mouse brain MAB2160 [21,22] cross-reacts with FXR1, and mAb7G1-1 cross reacts with caprin 1 [21,22,23] and argonaute [24].

FMRP and its murine homolog, FMRP, share 97% identity [19]. As in humans, various FMRP isoform bands ranging from 70 to 80 kDA were detected by western blot with MAB2160 by Khandjian et al. in 27 different mouse tissues [20]. The relative proportion of isoforms varied in different tissues and FMRP expression was higher in young mice. The relative amount of FMRP in diverse tissues was estimated by density scanning of the autoradiographs and arbitrarily assigning a value of 1 to the faintest band. FMRP was most abundantly expressed in brain and testis. Densitometric quantitation of FMRP signal on immunoblots (MAB2160) was also used to calculate the percentage of total FMRP present in various subcellular fractions of human lymphoblastoid cells [25]. The expression of FMRP in lymphoblastoid cell lines derived from PM and control males was assessed by Western blot using MAB2160 and an antibody against β-tubulin [26]. Upon densitometric scanning FMRP signals were normalized with β-tubulin and with the total loaded protein. FMRP levels in PMs were found to be not significantly different from those of control males. 

By contrast, Bagni’s group reported a reduction of FMRP in PM carriers. They used MAB2160 in Western blot with five lymphoblastoid lines derived from either control, PM or FM individuals and normalized FMRP to β-actin expression [27]. A similar approach was used by Kaufmann et al. to characterize FMRP with MAB2160 in peripheral leukocytes from a cohort of individuals comprising controls, PMs, FM females, and FM males with or without mosaicism [28]. The bands were quantified (optical density, OD) by scanning the autoradiogram and using image software. Several parameters were used to compare the protein level among samples including the (a) absolute OD; (b) ratio between FMRP of each subject and a normal control; and (c) ratio of the FMRP and β-tubulin values of each subject. Analysis done with the above parameters produced congruent results. Control and PM individuals of both sexes had the highest values, followed by FM females showing slightly lower levels. The lowest levels were found in FM males. Tassone and colleagues estimated FMRP levels in lymphocytes and fibroblasts of a cohort of 18 people—comprising FM, FM methylation mosaic, and FM size mosaic individuals by Western blot with MAB2160 [29]. FMRP levels decreased significantly with increasing methylation, and positively correlated with IQ scores. In both methylation and size mosaic FM males FMRP expression was 5.1-fold greater than that detected in FM.

FMRP levels in various human brain regions were reported by Fatemi et al. employing Western blots with rabbit anti-FMRP 17722 (Abcam, Table 1). The authors found that FMRP normalized to β-actin were reduced in the cerebellum from subjects with schizophrenia, bipolar disorders or major depression [30], and in the superior frontal cortex of people with autism [31]. The expression of FMRP in individuals with Alzheimer’s disease (AD) was analyzed using Western blot by Todd and colleagues [21]. FMRP levels were compared in post-mortem frontal cortex and cerebella samples of ten patients and ten controls using anti-FMRP rabbit antibody 17722 and an anti-β-tubulin antibody where no differences were found. The authors performed a similar study in a mouse double transgenic model of AD carrying two mutated human genes (APP, and presenilin 1). They compared FMRP levels—detected by Western blot with MAB2160—in cortical and cerebella lysates of control and AD double transgenic mice, and found no significant difference in expression.

Western blotting with mAb5C2 (Biolegend, San Diego, CA, USA; Table 1) was performed by Hampson and colleagues [32] to determine the levels of FMRP in the brain of *FMR1* KO mice following intracerebroventricular delivery of an adenovirus-associated vector carrying the *FMR1* gene into PND (post natal day) 5 pups. Injected mice and wild type (WT) controls were sacrificed either at PND 31 or 60, and various brain structures analyzed. The scanned bands were quantified and FMRP values normalized to glyceraldehyde 3-phosphate dehydrogenase (GAPDH) FMRP levels (percentage of the WT expression) were found to be 52% ± 9% in the hippocampus, 41% ± 13% in the striatum, and 71% ± 20% in the cerebral cortex in PND 31; and 47% ± 15% in the cerebral cortex, 48% ± 20% in the hippocampus and18% ± 5% in the striatum at PND 60.

Willemsen et al. [38,41,42] developed a knock-in (KI) mouse model of FXTAS (hereby referred as KI-mouse) carrying a 98 CGG repeats PM allele that demonstrated repeat instability on transmission and generated mice carrying alleles with differing CGG sizes. The levels of FMRP in brain samples of these mice were assessed by Western blotting with mAb2F5-1 (Table 1) [39]. Levels were normal in mice carrying repeats less than 110 CGG and were reduced to 5%–50% of the WT levels in those carrying expansions larger than 230 CGG repeats. These results, however, were obtained utilizing relatively small numbers of mice. 

To better understand the influence of CGG repeat length on the expression of FMRP, Hagerman and colleagues [43] measured and compared FMRP levels in whole brain extracts of larger cohorts of KI-mouse (*n* = 97) and WT (*n* = 57) mice. To avoid problems associated with protein precipitation and stability that could produce large errors in FMRP measurements, the authors processed the mouse brain according to a protocol that had been reported to solubilize 98% of brain tissues [44]. FMRP levels were assessed in western blot with MAB2166 and a chicken anti-GADPH antibody. Their analysis revealed that FMRP decreased with increased CGG repeat length throughout the PM range, and that at any given CGG length (normal and PM) there was a broad variation of FMRP levels. A similar Western blot analysis of FMRP levels in post-mortem mid-frontal cortex samples from seven control and 17 PM with FXTAS revealed a moderate reduction to 60% of control levels in the PM range of 56–118 CGG repeats [43]. 

Kumari et al. analyzed human fibroblasts from nine control individuals to quantify FMRP by immunoblot with MAB2160, and *FMR1* mRNA by quantitative reverse transcription polymerase chain reaction (qRT-PCR) [45]. They found that FMRP levels normalized to β-actin were variable and observed a significant negative correlation between FMRP and *FMR1* levels. Higher levels of FMRP were seen in fibroblasts from newborns confirming previous reports using dried blood spots (DBS) [33,40]. 

Lessard et al. quantified FMRP levels in blood platelet extracts from 1 mL of blood of 124 control and 26 FXS individuals using immunoblots with MAB2160 [34]. In samples from the control population the authors reported a normal distribution of FMRP values with a mean of 29.6 ± 7.5 pg/10^6^ platelets, and a range of 10 pg to 54.9-pg/10^6^ platelets. By contrast, no FMRP was detected in 12 out of 16 male FXS samples while the remaining four male FXS samples had low levels of FMRP, 8.6 pg/10^6^ platelets, probably due to mosaicism. In FM females FMRP levels were higher than in males, 11.1 to 27.3 pg/10^6^ platelets. A receiver-operating characteristic (ROC) curve analysis showed that at a threshold of 27.3 pg/10^6^ platelets, the assay had a sensitivity of 100% and a specificity of 61.3% for both genders. 

Some of the above studies detected FMRP levels in PM carriers that were not consistent. In particular, three reports found no difference in FMRP levels between PM carriers and control individuals [26,28,46], while others found a reduction of FMRP expression either in all PM carriers [7,27,43] or in those with low or high CGG repeat alleles [47]. The incongruence could (1) be caused the action of genetic and epigenetic factors responsible for the clinical phenotypes associated with PM; (2) stem from the way sample extraction [43] and Western blotting were performed; and (3) be caused by the lack of specificity of the anti-FMRP antibodies used. Background staining is obviously expected in immunoblots probed with mAb7G1-1, which is known to cross-react with two other proteins, caprin1 [21,22,23] and argonaute [24]; and with MAB2160—the antibody used in most Western blot experiments—which cross-reacts with FXR1 [22,45]. The MAB2160 epitope was mapped [35] to the sequence _34_NNWQPD_39_ of human FMRP, which is conserved except for the change of D_39_ to E in both mouse FMRP and in FXR1 (NNWQPE).

The Western blot procedure involves several steps from sample preparation, to sodium dodecyl sulfate polyacrylamide gel electrophoresis (SDS PAGE), protein blotting, selection of antibodies, incubation, detection methods and normalization. According to Taylor, unless all the above steps are rigorously performed according to an appropriately tested protocol, Western blots produce at best an approximate estimation of protein levels [48]. Establishment of reliable measurements requires determination of several parameters including the linear and quantitative range of both FMRP and the protein used for normalization, the efficiency of protein transfer onto the membrane, the optimization of protein loading for FMRP, and the housekeeping protein used as control. In the works discussed above FMRP was normalized using GAPDH, β-actin, or β-tubulin. These housekeeping proteins are constitutively expressed in high amounts in all cells, and are easily overloaded with the target protein upon loading a total amount of protein of 10–50 µg per gel lane. Taylor reported that in HeLa cells, endogenous GAPDH can be reliably quantified by loading no more than 0.5 µg of total protein per lane [48].

Aware of the limitation of Western blotting as a reliable protein quantification method, Warren and colleagues investigated the influence of CGG repeat number on the FMRP level by developing a more accurate assay for measuring the protein. They used a slot-blot-based immunoassay [7] to analyze lymphoblastoid cell lines from males carrying control, PM, or full mutation alleles. Several dilutions of each sample were blotted in duplicate onto nitrocellulose membranes, which were then incubated either with MAB2160 or an antibody against -eIF4E (eukaryotic translation initiation factor 4E). Dilutions of purified FMRP and eIF4E were also blotted to prepare standard curves that were used to calculate the molarity of both eIF4E and FMRP. FMRP values were normalized using eIF4E as protein control. A reduced level of FMRP was found in four PM males (22% reduction) carrying alleles ranging from 105 to 130 CGG repeats and in two males (17% reduction) with intermediate 48–55 repeats. A limitation of this study was the extremely small number of PM subjects (*n* = 6) and the broad variation of FMRP levels detected in both the control and PM samples. Sherman used the same slot-blot-based immunoassay [7] to quantify FMRP in blood samples of 74 PM male carriers [49]. In contrast with Warren’s group, they reported similar FMRP levels (FMRP/eIF4E) for mid-PM (80–89 CGG) carriers and controls (<55 CGG). Lower FMRP levels were found in PM males carrying either low (55–79 CGG) or high (90–120 CGG) PM alleles. 

Despite the inconsistent data on the FMRP levels detected in individuals carrying a PM allele, both Western blot and slot-blot immunoassays have firmly established that FMRP is either lacking or extremely reduced in FM males, and reduced in FM females. Moreover, the immunoassays have produced extremely valuable data on the expression of the protein and its isoforms in different tissues and developmental stages.

## 3. Immunohistochemistry

Immunohistochemical staining of tissues and cells has been extensively used to detect specific proteins for research and diagnostic purposes. The immunohistochemical detection of FMRP in human tissues was first reported by Mandel using MAB2160 [13]. Intense staining was found in the soma of neurons, in spermatogonia, and epithelial tissues. A method for the diagnosis of FXS by antibody detection of FMRP in blood smears was established using MAB2160 by Willemsen [50]. FMRP staining was observed in the cytoplasm of lymphocytes from control individuals and not detected in FM males. FMRP was also found in lymphocytes from PM carriers. 

The antibody test was evaluated by two independent laboratories using a cohort of 173 individuals including 113 FM patients [51]. Both laboratories utilized the same protocol with smears prepared immediately after blood sampling and stored at −80 °C. They examined 100 lymphocytes in every smear and scored for the presence of FMRP immunostaining. A lymphocyte was scored positively independently of the degree or intensity of the staining, and the number of stained cells was reported as percentage of the total lymphocytes examined. The mean percentage of stained lymphocytes in control females and males were not significantly different, 80% (*n* = 27, (standard deviation) SD = 6) and 89% (*n* = 33, SD = 9), respectively. A much lower mean percentage was obtained in affected FM males (7%; *n* = 69, SD = 7) and FM females (39%, *N* = 44, SD = 19). The authors detected variability in the number of FMRP-stained cells in both control and FM individuals. In males, the distributions of the percentage of FMRP-stained lymphocytes in control and FM individuals had completely separated values. By contrast, in female the two corresponding distributions had partially overlapping values. Thus, the antibody test was a much more specific diagnostic tool in discriminating between control and FM males than in control and FM females. The test was rapid, easy to run, inexpensive, and could be used to analyze a large number of samples and was successfully utilized to screen 412 males with severe developmental disabilities of unknown cause allowing the detection of two FM individuals [52]. 

To study the relationship between FMRP expression and the various clinical, cognitive, and adaptive-skill features associated with FXS, Taylor and colleagues utilized this antibody test to screen a cohort of 80 FXS individuals comprising fully methylated FM males and females; FM mosaic males with a PM; and partially methylated FM males [53]. The percentage of positively stained lymphocytes correlated with the number of FXS physical features; it also correlated with IQ (intelligence quotient) in males with partially methylated FM and in FM females. Low percentage of FMRP expressing lymphocytes was reported in PM males carrying alleles with more than 100 CGG repeats [54]. The antibody test was used to study the expression of FMRP in blood smears of 55 autistic boys [55]. In this population, the authors reported very low levels of FMRP with an average percentage of stained lymphocytes of 8.6 per child, and no autistic behavior/FMRP interaction. The assay was also employed to study FMRP expression in a Mexican cohort comprising FXS patients and control individuals [56]. The authors reported that the sensitivity and specificity of the assay and the positive and negative predictive values were 100%. In all the above reports, the FMRP level was gauged by determining the percentage of stained lymphocytes in the sample and the fraction of positively stained cells used to assess FMRP levels. Thus, in this assay weakly stained lymphocytes were weighted the same as heavily stained one. This test made possible for the first time to screen directly for FMRP and allowed to distinguish FM males from control individuals with high discriminating power. However, the assay was less specific in discriminating FM females from control individuals.

Willemsen adapted the immunohistochemical test for prenatal diagnosis of at-risk male fetuses using chorionic villi at 12.5 weeks of gestation [57,58]. A high amount of FMRP was detected in the cytotrophoblasts of chorionic villi of control fetuses. None was found in the villi of affected fetuses. The test was also employed in the prenatal diagnosis of FXS using uncultured amniotic cells or blood from fetuses [51,59]. 

The same group also devised a non-invasive immunohistochemical test for the detection of FMRP in hair roots [60]. In the assay, hair roots (10 to 20 hairs) with visible bulbs were fixed, processed for immunostaining with the anti-FMRP antibody MAB2160, (Table 1) and examined with a stereo microscope for the presence or absence of FMRP in bulb cells. The number of positively stained hair roots was then expressed as percentage. In control individuals FMRP staining was detected in most hair roots (77% to 100%). Most of the hair roots from FM males had no staining; however, in 45% of the FM males a low percentage (<30%) of roots were stained. According to the authors, the percentage of the latter group suggested that the samples could represent males with a mosaic genotype at DNA level. The percentage of stained roots in affected FM females was variable ranging from 0%–55%. The expression of FMRP in a FXS family was studied using both the blood smear and the hair root assays [61]. It was found that the percentage of stained roots was a better indicator of cognitive functioning than the percentage of labeled lymphocytes. The hair root assay was used to screen for FM patients among a population of 300 males with developmental disabilities enrolled in a special education school. The test identified all FM males (five) present in the cohort [62] and according to the authors was highly appropriate for screening of male populations. All the immunochemistry assays described above were rapid and made possible for the first time the diagnosis of FXS by screening directly for FMRP.

## 4. Capture Immunoassay

Capture (sandwich) immunoassays detect analytes with high sensitivity and specificity because they require the binding of two specific antibodies to the molecule of interest. The development of such immunoassays for the direct quantification of FMRP has been hampered by the lack of high affinity anti-FMRP antibodies capable of binding efficiently to the protein. To explore the correlation between FMRP levels and the various clinical phenotypes associated to the diverse *FMR1* genotypes, Iwahasi [36] developed a sandwich Enzyme-Linked ImmunoSorbent Assay (ELISA) to quantify FMRP in non-transformed peripheral lymphocytes. Since the anti-FMRP antibodies available to their laboratory lacked the avidity necessary to capture FMRP in lymphocyte extracts, the authors obtained a high affinity chicken polyclonal antibody using a peptide sequence located at the carboxyl-end of FMRP as the immunogen. The antibody specificity and avidity was characterized in Western blots and compared to that of MAB2160. The polyclonal antibody strongly bound to several FMRP isoforms and cross-reacted with a non-identified high molecular weight band. Since this band was not detected by MAB2160, it was concluded that the combination of the polyclonal antibody and MAB2160 would detect, specifically, FMRP. 

**Figure 1:**
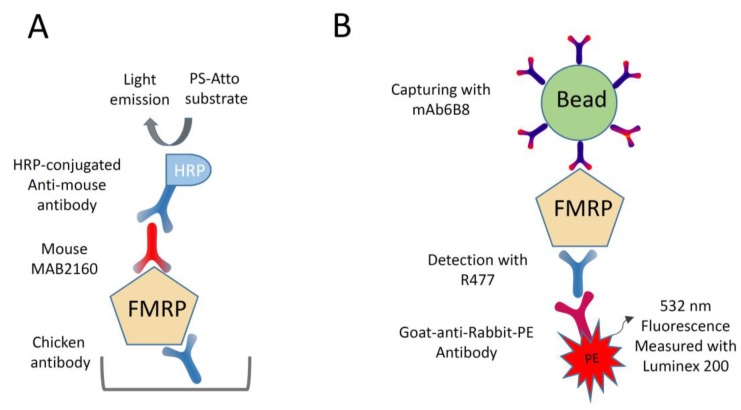
Capture immunoassays for Fragile X Mental Retardation Protein 1 (MRP) quantification. (**A**) Chemilunescent sandwich Enzyme-Linked ImmunoSorbent Assay (ELISA). FMRP was captured by the anti-FMRP chicken antibody and detected by the mouse monoclonal antibody MAB2160 (Table 1). After incubation with horseradish peroxidase-conjugated anti-mouse Ig (HRP), FMRP levels were assessed in a luminometer by measuring light emitted from the horseradish PS-Atto substrate; and (**B**) Luminex-based capture immunoassay. FMRP was captured by mAb6B8-coated beads and detected by the rabbit antibody R477. After incubation with a goat anti-rabbit Immunoglobulin G (IgG) conjugated to phycoerythrin (PE), FMRP levels were assessed with a Luminex 200 system by measuring PE fluorescence.

The chicken antibody was employed in several sandwich-ELISA protocols either as detecting or capturing antibody, utilizing a number of peroxidase substrates and detection techniques. Chemiluminescent ELISA detection in a luminometer with PS-Atto as substrate, allowed the authors to establish a protocol capable of quantifying FMRP using the chicken polyclonal as the capturing antibody and MAB2160 as the detecting antibody (Figure 1A). The ELISA was run in 96-well-plates that had been coated from 24 to 48 h, and required 36–48 h to complete. The FMRP levels in lymphocyte extracts from FM, mosaic and control males, was measured using the assay. A maltose-binding fusion protein (MB-FMRP) containing the whole FMRP sequence was constructed and evaluated as a possible ELISA standard. It was found, however, that the protein was not stable in solution due to its tendency to precipitate upon long storage. Consequently, the levels of FMRP in FM and mosaic males were reported as a percentage of the mean levels calculated for control males. The range of FMRP measured in 6 FM was from 0.48% to 4.45% of the mean levels found in 15 controls; the levels detected in eight mosaic males were from 1.07 to 13.02%. The difference between FM and mosaic was not statistically significant. However, the differences between FM and control and mosaic and control were both statistically significant. The ELISA was used by the same group [63] to measure FMRP levels in peripheral lymphocytes of 23 PM males. It was found that FMRP was reduced by 12% in the PM males compared to the mean levels in male controls. 

In the ELISA [36,63] it was critical to capture with the chicken antibody; capturing with MAB2160 failed to produce signals above the background. Independently of the order of antibody used, a low signal to background ratio made it not possible to utilize alternative peroxidase substrates. Unacceptable background excluded the use of several blocking solutions except for hydrolyzed casein. However, even with this agent the high background could be suppressed only by delaying its application to the second step and performing the capture step in absence of blocking solution. The authors concluded that the chicken antibody had the avidity necessary to bind to FMRP in a complex mixture such as lymphocyte extracts but was somehow unable of capturing the protein in the presence of any blocking agents. The chicken polyclonal antibody allowed for the first time the establishment of an accurate capturing immunoassay for FMRP. Caveats of this ELISA were the use of bona-fide normal controls as standard that limited the possibility of comparing data from different laboratories, and the long incubation times required by several steps of the protocol.

In a quest to obtain anti-FMRP monoclonal antibodies possessing the high affinity and avidity necessary for binding and capturing the protein in cell extracts, our group has developed anti-FMRP mAbs using recombinant FMRP expressed in insect cells infected with baculoviruses harboring the entire *FMR1* open reading frame [33]. One antibody, mAb6B8 (Table 1) specifically reacted with high affinity to full-length human FMRP P (Biolegend, San Diego, CA, USA) [33]. An anti-FMRP polyclonal antibody, R477 (Table 1) was obtained by immunizing rabbits with a synthetic oligopeptide, DDHSRTDNRPRNPREAK spanning amino acid residues 554–570 of FMRP [33]. Western blot analysis indicated that R477 reacted specifically to full-length FMRP. The two antibodies, mAb6B8 and R477 were evaluated for their capacity to capture and detect FMRP in Luminex-based capture immunoassays. One antibody was coupled to xMAP MicroPlex microspheres (Luminex, Austin, TX, USA) and used to capture FMRP—and the other to detect it.

An assay capable to quantify FMRP was established by capturing the protein with mAb6B8 and detecting it with R477 (Figure 1B). The Luminex assays were prepared in low-protein-binding Durapore MultiScreen 96-well filter plates (EMD Millipore, Billerica, MA, USA) and run according to a multistep protocol that required 24 to 36 h. In each well a sample (50 µL) was step-wise incubated with xMAP MicroPlex microspheres coupled with mAb6B8, R477, and goat anti-rabbit IgG conjugated to phycoerythrin. Finally, microspheres were resuspended in 100 µL assay buffer and the median fluorescence intensity (MFI) measured using a Luminex 200 system (Luminex Corporation, Austin, TX, USA). The assay was evaluated using 1–80 µg of protein extracted from control and full mutation male lymphoblastoid cell lines. The level of FMRP (MFI) in control cells was a function of the amount of sample and a linear response was detected up to 40 µg of extract (*R*^2^ = 0.98). Only background fluorescence values were detected with 1–80 µg of male FM extracts. 

The assay was also performed using freshly prepared lymphocytes and dried blood spots. Repeated assays with extracts of 19 lymphocytes from controls were highly correlated (*r* = 0.96), indicating that the assay was reliable. A broad FMRP distribution was found in 11 lymphocytes from controls; MFI ranged from 1194–2375 (SD, 405.1). A lymphocyte extract from a FM male showed only background fluorescence value and was easily distinguished from control samples. Since it had been reported that both recombinant FMRP and MB-FMRP fusion proteins were not reliable as standards for absolute quantification because they were unstable in solution and produced insoluble aggregates [36], we engineered an abbreviated FMRP fusion protein, GST-SR7 carrying only short domains of FMRP attached to the recombinant protein, glutathione S-transferase (GST). This protein carried the binding sites of both mAb6B8 and R477, was stable upon storage at −70 °C, did not form aggregates, and was reliable as a standard protein [33]. In the assay, a linear response was obtained using GST-SR7 at a concentration of 0.5 to 280 pmol/L. Using mAb6B8, R477 and the GST-SR7, we developed a rapid (24–36 h), high sensitive methods for quantifying FMRP from dried blood spots (DBS), lymphocytes and other human tissues. DBS are blood samples blotted and dried onto filter paper that can be easily stored and transported at room temperature for analysis. Since their introduction by Guthrie in 1963, DBS have been used to screen for metabolic diseases in large populations of newborns [64]. We measured FMRP levels in DBS (6.9 mm-diameter disks) from 215 individuals carrying either control, PM, or FM alleles. FMRP was reported as concentration (pmol/L) in the 50-µL extracts used in the assays which were prepared from eluting three disks in 200 µL buffer—corresponded to 8.7 µL of whole blood. As with lymphocytes, assays of duplicate extracts of 57 randomly selected DBS were highly correlated (*r* = 0.96), indicating that the assay was consistent. In control individuals, FMRP levels followed a normal distribution (mean 25.9 pmol/L, SD 9.6 pmol/L), similar to that reported by others in platelets using Western blot [34]. In adults, gender and age had no effect on FMRP expression. However, in infants and preteens there was a higher amount of FMRP which was inversely correlated to age and decreased to adult levels in teenagers. We speculated that since DBS disk samples corresponds to a specific volume of blood and FMRP is expressed prevalently in leukocytes, the gradual decrease of FMRP from infants to teens could be caused by the reduction seen in leukocyte count in children as they get older. 

In males with a FM allele, the mean FMRP level was 1.7 pmol/L (6% of control), with a maximum of 6.6 pmol/L (26% of control). The FMRP values found in FM and control individuals were not overlapping, and according to ROC analysis, at a cutoff of 7.59 pmol/L sensitivity and specificity were both 100%. The assay identified all the 17 FM males in the population and distinguished between mosaic (mean 3.3 pmol/L; SD = 1.6 pmol/L) and non-mosaic FM samples (mean 0.6 pmol/L; SD = 0.3 pmol/L). In FM females, the FMRP expression (mean 17.2 pmol/L) was significantly different (*p* = 0.032, U-test) from that detected in control females (mean 26.0 pmol/L). No significant difference in FMRP levels was detected between PM and control females (mean 23 pmol/L, and 26 pmol/L, respectively). Our unpublished data show that the variability of FMRP values detected in a cohort of 40 control individuals is substantially decreased upon normalization to the number of leukocytes in the DBS samples. Thus, using FMRP values adjusted to the leukocyte counts could ameliorate the discriminatory power of the assay in distinguishing between affected FM females and control females. In as much as DBS requires just a few drops of blood on a card and can be stored and transported at room temperature, the possibility of running the Luminex assay on DBS greatly expanded its use, making it possible to analyze samples collected in several laboratories. The assay was accurate, economic, easy to run, rapid, and amenable to high-throughput analysis, and could be an effective method for screening of newborns and other populations.

In order to characterize FMRP levels in a neonate population which is a potential target for screening with the Luminex-based immunoassay [33], we screened 2000 randomly-selected residual DBS [40] from a state-mandate newborn screening for metabolic and genetic diseases (Wadsworth Center, New York State Department of Health, Albany, NY, USA). The DBS disks (duplicate—3 mm diameter, 7.1 mm^2^) were from anonymous newborn females (1000) and males (1000) that had been properly stored with a desiccant in a refrigerator for five weeks. FMRP levels in each disk was assessed using GST-SR7 as a standard [33,40]. The results showed a broad distribution of FMRP ranging from 10.3- to 92-pmol/L, with a mean FMRP of 44.8 pmol/L, and a SD of 12.4 pmol/L. When compared to FMRP in adults (done using 6.9 mm-diameter DBS disks, 37.4 mm^2^) [33], the mean concentration of FMRP in neonates (3 mm-diameter disks) was about seven-fold higher (6.3 pmol/L eluted per mm^2^) than in adults (0.93 pmol/L eluted per mm^2^).

In order to determine what *FMR1* alleles were present at the low end of the FMRP distribution, we analyzed the CGG region of the fourteen samples that had FMRP levels lower than two SDs below the mean [40]. Thirteen samples had normal alleles and their reduced levels of FMRP were caused by factors other than the *FMR1* genotype. In contrast, one sample from a female had a reduced FMRP expression because of a large 161/167 CGG-repeat PM allele, which was shown by methylation analysis to reside on the active X chromosome in 90% of the leukocytes. No FM males were found in the limited number of DBS used in this study. However, the assignment of the sample from a female with a 161/167 CGG-repeat allele to the extreme low range of the FMRP distribution showed that Luminex assay had high discriminatory power.

The assay was also applied to quantify FMRP in 74 DBS from the New South Wales (Australia) newborn screening program that had been stored up to seven years, and included samples from 68 control newborns and six FM males [40]. This study was performed as a blinded experiment in order to assess whether FMRP levels correlated with the diagnoses of FXS syndrome that had been made by the GOLD Service Hunter Genetics (Newcastle, Australia). The long storage time resulted in the decrease of measurable FMRP that was extremely reduced in DBS stored four years or longer. The mean of the samples from control newborns—stored up to four years—was approximately one third of the mean of newborn fresh DBS. Notwithstanding the diminution of FMRP, the assay identified all six samples from FM males among the 74 aged DBS. We showed, for the first time, that it was possible to accurately quantify FMRP in 3-mm-diameter newborn DBS disks which are used for state mandatory screening, and suggested that the Luminex immunoassay could serve as the first step in FXS screening of newborns and in at risk populations. Moreover, the high sensitivity of the assay allowed distinguishing FM males from control newborns using DBS that were stored up to four years.

## 5. Time-Resolved Fluorescence Resonance Energy Transfer Immunoassays

**Figure 2:**
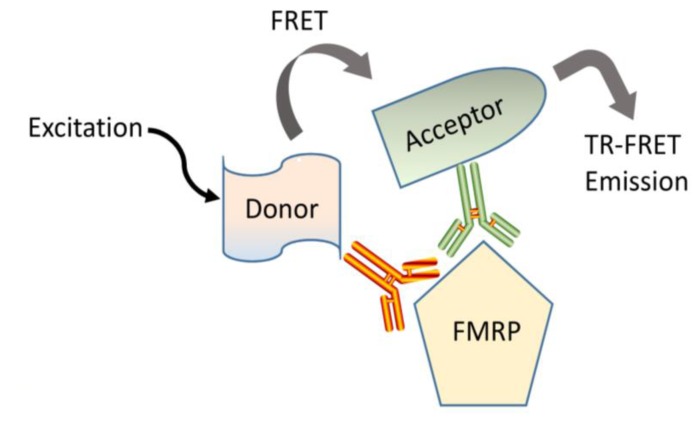
FMRP time-resolved fluoresce resonance energy transfer (TR-FRET) immunoassays. Binding of two antibodies to proximal domains of FMRP allowed the donor dye (europium cryptate or Lumi4-Tb) attached to one antibody to transfer resonance energy to the acceptor dye (d2) coupled to the second antibody generating a unique detectable fluorescent energy that was assessed using an Envision Reader (Perking Elmer Inc., Waltham, MA, USA).

Two laboratories have reported the development and characterization of assays that employ time-resolved fluorescence resonance energy transfer technology (TR-FRET) to quantify FMRP [8,35]. These immunoassays utilize the simultaneous binding of a pair of fluorophore-labeled antibodies recognizing two close epitopes of the target protein. One antibody carries a donor dye (europium cryptate or Lumi4-Tb cryptate) capable to be excited and transfer resonance energy to the acceptor dye d2 attached to the second antibody (Figure 2). Once the donor and acceptor fluoropheres are brought in close proximity to each other by the antibodies’ binding, a unique detectable fluorescent signal is generated. These immunoassays require small amount of sample, are run by incubating the labeled antibodies simultaneously to the analytes, involve simple one-step protocols that do not require separation steps, and are suitable to micro-plate high-throughput screening [65].

Weiss established FMRP TR-FRET assays using two couples of antibodies [35]. They labeled MAB2160 with the donor fluorophore Lumi4w-Tb, and the rabbit antibody F4055 i(Sigma, St. Louis, MO, USA; Table 1) or mAb3E11 (Table 1) with the acceptor fluorophore d2. Week specific fluorescent signals were detected in the first assay in which the recombinant MBP-FMRP fusion protein was incubated with MAB2160-Tb and F4055-d2. Stronger specific signals were detected in the second assay with antibodies MAB2160-Tb and mAb3E11-d2 which bind to epitopes located both in the amino region of FMRP. The FMRP specific energy transfer (delta F) was calculated as the ratio of the acceptor d2 (665 nm) and the donor Tb (620 nm) emission of the sample normalized to the background (d2/Tb ratio calculated for the buffer) and expressed as percentage. Both assays were optimized for the amount of donor and acceptor antibodies per well, and a serial dilution of MBS-FMRP was used to assess the dynamic range, lower limit of detection and intra-assay and inter-assay stability. A dynamic range of three orders of magnitude, up to 2 ng/µL, and a low limit of detection of 40 pg/µL (400 pmol/L) or 10-pg/µL (100 pmol/L), were reported for the first and second assay, respectively. The second assay was optimized for detection of FMRP in cells using HEK293 cells transfected with a plasmid expressing human FMRP or a mock plasmid. Next, lysates of fibroblasts derived from FXS patients were spiked with different amounts of recombinant FMRP and used to establish the linearity of the assay in a complex cellular system. Endogenous FMRP was detected in cultured fibroblasts derived from control individuals and not in fibroblasts of FXS patients. FMRP levels in peripheral blood mononuclear ear cells from control individuals were 6–7 times higher than those prepared from FXS patients. The assay had an elevated low limit of detection (10 pg/µL; 100 pmol/L) compared to a TR-FRET immunoassay for the detection of α-synuclein (0.1 ng/mL; 6.9 pmol/L) reported previously by the same group [65], and was not used for high-throughput screening of FMRP.

Recently, Usdin devised similar FMRP TR-FRET assays and used one of them for high-throughput screening to identify compounds that increase the expression of FMRP in cultured neuronal stem cells [8]. The assays were established using five anti-FMRP antibodies—developed by CisbioUS (Bedford, MA, USA)—each binding to a distinct epitope. The antibodies were labeled with either europium cryptate (K, donor) or the d2 acceptor, and different pairs were assembled by combining a donor-labeled antibody (Ab-K) with an acceptor, Ab-d2. The pairs were tested with lysates prepared from fibroblasts of control and FXS individuals. A pair of anti-FMRP antibodies (Table 1), clone 2D4 labeled with the K-donor (2D4-K) and clone D14-F4-labelled with the d2-acceptor (D14-F4-d2), showing the highest signal to background ratio was evaluated using two-fold dilutions of a recombinant FMRP in 384-well plates, where it showed a linear response between 4 fmol/µL (4 nmol/L) and 270 fmol/µL (270 nmol/L) FMRP. This assay was optimized for lymphoblatoid cells or fibroblasts derived from control and FXS individuals. Upon incubation of the labeled antibodies with 2500 cells per well in the 1536-well plates, FMRP levels in control cells were four to five-fold higher than those of FXS cells. The assay was also appropriate to detect FMRP in neurons and neuronal stem cells (NSCs). This assay was used with FXS-patient derived fibroblasts to screen a compound library (LOPAC^1280^) for chemicals capable of increasing FMRP levels. The screening had a signal to basal ratio, S/B, of 4.3-fold, a coefficient of variation, CV, of 4.3%, and a Z factor, Z, of 0.70; and identified one hit compound, protoporphirin (PPIX). Screening of LOPAC^1280^ with FXS derived NSCs and neurons, identified as hit compounds PPIX, and SB216763, which were shown by qRT-PCR to increase also the level of *FMR1*. A larger TR-FRET screening was performed using FXS-derived NSCs and a FDA-approved drug library containing about 4000 compounds. Four of the hit compounds, sodium decanehydroxamate, geliomycin, tibrophan, and deserpidine were also positive in qRT-PCR (increased *FMR1*). However, Western blot analysis of FXS NSCs treated with these compounds FMRP did not detect FMRP. The authors concluded that the drugs had a modest effect in FMR1 activation, and that additional compound libraries should be screened to identify drugs that reactivate FMR1 expression more effectively.

In as much as the TR-FRET platform allows the design of specific, sensitive immunoassays that can be used in a variety of high-throughput applications, both the Weiss’ assay [35] and the Usdin’s assay [8] showed elevated low limit of detections of FMRP (100 pmol/L, and 4 nmol/L, respectively) and low signal to basal ratios (seven- and 4.3-fold, respectively) that do not compare well with those reported by us [33,40] with a capture Luminex-based immunoassay for FMRP (0.5 pmol/L; S/B of 7.8- and 42-fold, respectively for mosaic and non-mosaic FM males). To maximize the resonance energy transfer between donor and acceptor fluoropheres, anti-FMRP antibodies that bind with higher affinity to the protein should be used to design new TR-FRET assays.

## 6. Concluding Remarks

The literature we have reviewed describes the use of diverse approaches to either estimate or quantify FMRP for research and/or diagnosis. Western blotting has been used to characterize the expression of the protein in tissues and developmental stages. This approach has been instrumental in determining the localization of the protein in the cells and in tissues in both human and mouse. It has been firmly established that the lack, or extreme reduction, of FMRP expression is the cause of FXS. It remains to be determined whether or not there is a reduction of FMRP levels in some carriers of PM alleles. Immunohistochemical staining for the detection of FMRP in lymphocytes, cytotrophoblasts of chorionic villi, uncultured amniotic cells, or hair bulbs has been successfully used for the rapid diagnosis of FXS. The development of high affinity antibodies against the protein has made possible the use of sandwich immunoassays for the accurate quantification of FMRP. Both the chemiluminescent ELISA [36] and the Luminex-based immunoassay [33] have been successfully applied to distinguish FM patients from control individuals. The latter assay uses an abbreviated FMRP-fusion protein for the precise quantification of FMRP and can be used for detecting the protein in the DBS. It distinguishes non-mosaic and mosaic males from control individuals with sensitivity and specificity approaching 100% and has been used to screen 2000 newborn residual DBS samples [40]. FMRP TR-FRET assays have been used for high-throughput screening of cultured neuronal stem cells to identify compounds that increase the expression of the protein [8]. The development of these methods now allows the rapid diagnosis of FM males lacking or having reduced FMRP in blood samples including DBS.

## Figures and Tables

**Table 1:** Anti-Fragile X Mental Retardation Protein 1 (FMRP) antibody used in the cited literature.

Antibody	Species Specificity	Immunogen	Cross Reactivity	Reference
***Monoclonal antibodies***				
MAB2160 (mAb1C3, mAb1A)	m, h, r	Rec. FMRP (C17 clone)	FXR1	[13,18,19,20,21,22,23,24,25,26,27,28,29,30,31,32,33,34,35,36]
mAb7G1-1	m	aa354–368	Caprin 1, AGO	[18,21,22,23,24,37]
mAb6B8	h	Rec. FL FMRP	No	[23,27]
mAb5C2	m, h	Rec. FL Fmrp	No	[32]
mAb3E11	h	N-end	NA	[35]
clone 2D4	h	NA	NA	[8]
clone D14-F4	h	NA	NA	[8]
mA2F5-1	h, m	N-end	NA	[38,39]
***Polyclonal antibodies***				
Chicken Ab	h	C-end	high MW band	[36]
R477 Ab	h, m, r	C-end	weak 65 kDa band	[33,40]
R-F4055	h	C-end	NA	[35]
α765	h	aa314–443	NA	[14]
Ab17722 (Abcam)-R	h, m, r	C-end	FXR1	[21,30,31]

Legend: m—mouse, h—human, r—rat, R—rabbit, Rec.—recombinant, FL—full length, NA—not available.
